# Targeting HIV/HCV Coinfection Using a Machine Learning-Based Multiple Quantitative Structure-Activity Relationships (Multiple QSAR) Method

**DOI:** 10.3390/ijms20143572

**Published:** 2019-07-22

**Authors:** Yu Wei, Wei Li, Tengfei Du, Zhangyong Hong, Jianping Lin

**Affiliations:** 1 State Key Laboratory of Medicinal Chemical Biology, College of Pharmacy and Tianjin Key Laboratory of Molecular Drug Research, Nankai University, Haihe Education Park, 38 Tongyan Road, Tianjin 300353, China; 2 Platform of Pharmaceutical Intelligence, Tianjin International Joint Academy of Biomedicine, Tianjin 300000, China; 3 State Key Laboratory of Medicinal Chemical Biology, College of Life Sciences, Nankai University, 94 Weijin Road, Tianjin 300071, China; 4 Biodesign Center, Tianjin Institute of Industrial Biotechnology, Chinese Academy of Sciences, Tianjin 300308, China

**Keywords:** HIV/HCV coinfection, machine learning, multiple QSAR method, multitarget inhibitors, polypharmacology, drug discovery

## Abstract

Human immunodeficiency virus type-1 and hepatitis C virus (HIV/HCV) coinfection occurs when a patient is simultaneously infected with both human immunodeficiency virus type-1 (HIV-1) and hepatitis C virus (HCV), which is common today in certain populations. However, the treatment of coinfection is a challenge because of the special considerations needed to ensure hepatic safety and avoid drug–drug interactions. Multitarget inhibitors with less toxicity may provide a promising therapeutic strategy for HIV/HCV coinfection. However, the identification of one molecule that acts on multiple targets simultaneously by experimental evaluation is costly and time-consuming. In silico target prediction tools provide more opportunities for the development of multitarget inhibitors. In this study, by combining Naïve Bayes (NB) and support vector machine (SVM) algorithms with two types of molecular fingerprints, MACCS and extended connectivity fingerprints 6 (ECFP6), 60 classification models were constructed to predict compounds that were active against 11 HIV-1 targets and four HCV targets based on a multiple quantitative structure–activity relationships (multiple QSAR) method. Five-fold cross-validation and test set validation were performed to measure the performance of the 60 classification models. Our results show that the 60 multiple QSAR models appeared to have high classification accuracy in terms of the area under the ROC curve (AUC) values, which ranged from 0.83 to 1 with a mean value of 0.97 for the HIV-1 models and from 0.84 to 1 with a mean value of 0.96 for the HCV models. Furthermore, the 60 models were used to comprehensively predict the potential targets of an additional 46 compounds, including 27 approved HIV-1 drugs, 10 approved HCV drugs and nine selected compounds known to be active against one or more targets of HIV-1 or HCV. Finally, 20 hits, including seven approved HIV-1 drugs, four approved HCV drugs, and nine other compounds, were predicted to be HIV/HCV coinfection multitarget inhibitors. The reported bioactivity data confirmed that seven out of nine compounds actually interacted with HIV-1 and HCV targets simultaneously with diverse binding affinities. The remaining predicted hits and chemical-protein interaction pairs with the potential ability to suppress HIV/HCV coinfection are worthy of further experimental investigation. This investigation shows that the multiple QSAR method is useful in predicting chemical-protein interactions for the discovery of multitarget inhibitors and provides a unique strategy for the treatment of HIV/HCV coinfection.

## 1. Introduction

Human immunodeficiency virus type-1 (HIV-1) is the causative factor of acquired immunodeficiency syndrome (AIDS), a pandemic disease [1]. In addition, hepatitis C virus (HCV) infection causes acute and chronic liver disease, including cirrhosis and hepatocellular carcinoma [2]. Unfortunately, since HIV-1 and HCV have similar routes of transmission, the risk of HIV/HCV coinfection is very high [3]. According to the World Health Organization (WHO), approximately 2.3 million of the estimated 36.7 million people living with HIV globally had been infected with HCV as of 2015. It is necessary for these people to be diagnosed and provided with effective and reasonable treatment for both HIV and hepatitis as a priority [4]. However, in the treatment of HIV/HCV coinfection, special considerations must be made for potential drug–drug interactions and toxicities. The standard treatment for HIV-1 is highly active antiretroviral therapy (HAART), which improves the quality of life of AIDS patients by reducing viral load [5]. HAART utilizes a cocktail of drugs to inhibit multiple viral proteins involved in the viral life cycle, including reverse transcriptase, integrase, and protease [6]. Although it is effective, HAART requires the sequential administration of single-target drugs that may result in drug–drug interactions, poor treatment adherence and the emergence of drug resistance. The current therapy for HIV/HCV coinfection includes regular injections of pegylated α-interferon (PEG-IFN) plus daily ribavirin (RBV) in combination with HAART [7]. As with the HARRT treatment regimen, additive drug toxicity, high cost and the long-term therapy cycle should be taken into account during HIV/HCV coinfection treatment. To avoid these undesirable factors, multitarget therapy using a single drug capable of simultaneously inhibiting two or more viral targets has been proposed to reduce the complexity of treatment [5].

**Figure 1:**
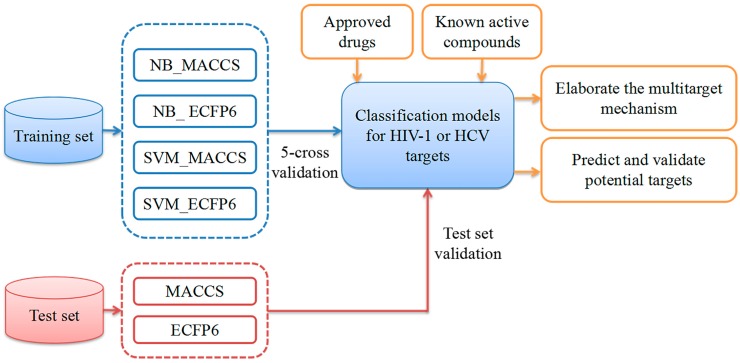
Flowchart showing the process of generating multiple quantitative structure–activity relationships (multiple QSAR) models for human immunodeficiency virus type-1 (HIV-1)/hepatitis C virus (HCV) coinfection (the blue, red and orange objects represent the phases of construction, validation and application of multiple QSAR models, respectively).

As a result of the development of computer-aided drug design (CADD) technology, several in silico methods have been exploited to predict chemical-protein interaction (CPI) pairs between drugs and target proteins [8,9,10,11,12,13,14]. These methods can be approximately classified into two categories: ligand-based [15,16,17] and structure-based [18,19]. Conventional quantitative structure-activity relationships (QSAR) methods generally use a group of analogs against one target to elucidate the relationship between chemical structure and biological activity. In 2009, Vina et al. developed a multitarget quantitative structure–activity relationships (mt-QSAR) classification model to predict the probability of drug binding for more than 60 different receptors based on molecular connectivity and receptor sequences [20]. In 2014, Fang et al. generated 100 mt-QSAR models for 25 key targets for the treatment of Alzheimer’s disease (AD) to predict the chemical–protein interactions and discover highly potent multitarget-directed ligands (MTDLs) [21]. In their research, the goal of mt-QSAR was to solve multiple binary classification problems instead of solving multilabel classification problems. In 2017, Antanasijević et al. proposed a regression multitasking quantitative structure–activity relationships (rmtk-QSAR) model based on the mt-QSAR methodology for the simultaneous prediction of the anticonvulsant activity and neurotoxicity of succinimides [22]. Hence, the mt-QSAR method has played a key role in the rational design of drugs for different biological targets. In this study, a multiple QSAR method intended for constructing multiple binary classification models was applied to predict the chemical–protein interactions (CPIs) for 11 key targets related to HIV-1 and four targets related to HCV. The workflow is depicted in Figure 1. First, by combining two machine learning algorithms (Naïve Bayes (NB) [23,24] and the support vector machine (SVM) [25]) and two molecular fingerprinting methods (MACCS [26] and extended connectivity fingerprints 6 (ECFP6) [27]), we developed multiple QSAR models for identifying inhibitors against 11 HIV-1-related and four HCV-related targets. Second, five-fold cross-validation and test set validation were used to evaluate the performance of all models. Third, to verify the application of this strategy, the multiple QSAR models that were developed were employed to predict the CPI for 27 approved HIV-1 drugs and 10 approved HCV drugs, as well as nine compounds known to be active against one or more targets of HIV-1 or HCV. The predicted results showed 20 hits to be potential HIV/HCV coinfection multitarget inhibitors, which were further confirmed by the assessment of bioactivity, resulting in seven compounds that actually interacted with HIV-1 and HCV targets simultaneously with diverse binding affinities. Our study indicates that machine learning-based multiple QSAR approaches could potentially be applied to the discovery of HIV/HCV co-inhibitors and other multitarget drugs.

## 2. Results

### 2.1. Experimental Dataset Analysis

**Figure 2:**
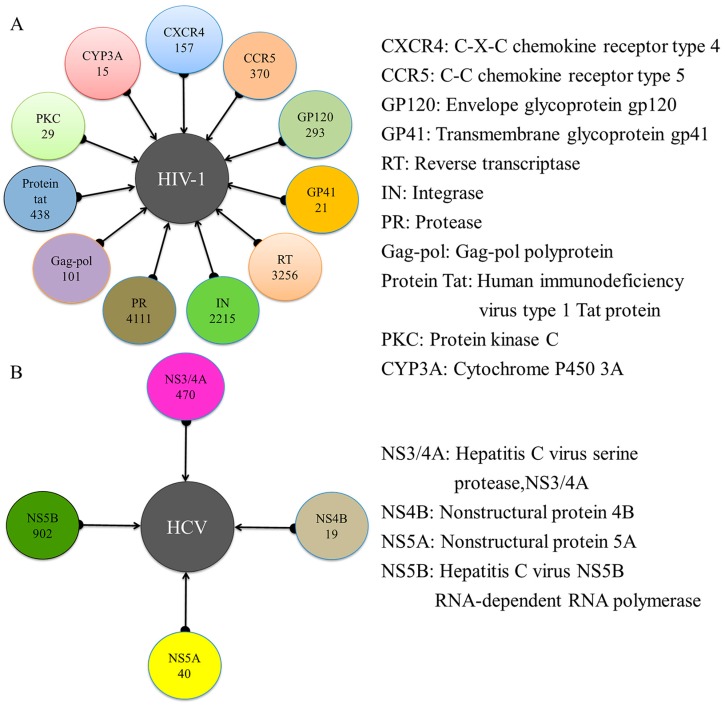
Overview of the targets related to HIV-1 (**A**) and HCV (**B**) and the total number of active compounds identified for each target.

In this study, a total of 11 targets related to HIV-1 and four targets related to HCV were obtained from the Therapeutic Target Database (TTD) [28] and ChEMBL Database (version 23, https://www.ebi.ac.uk/chembl/). The numbers of known inhibitors retrieved from the ChEMBL Database [29] that act on HIV-1 and HCV targets were 11,006 and 1431, respectively. The number of active compounds for each target is shown in Figure 2. A total of 11 datasets were generated for the HIV-1 system, including 157 inhibitors and 471 decoys for C-X-C chemokine receptor type 4 (CXCR4), 370 inhibitors and 1110 decoys for C-C chemokine receptor type 5 (CCR5), 293 inhibitors and 879 decoys for envelope glycoprotein gp120 (GP120), 21 inhibitors and 63 decoys for transmembrane glycoprotein gp41 (GP41), 3256 inhibitors and 9768 decoys for reverse transcriptase (RT), 2215 inhibitors and 6645 decoys for integrase (IN), 4111 inhibitors and 12,333 decoys for protease (PR), 101 inhibitors and 303 decoys for Gag-pol, 438 inhibitors and 1314 decoys for the protein Tat, 29 inhibitors and 87 decoys for protein kinase C (PKC), and 15 inhibitors and 45 decoys for cytochrome P450 3A (CYP3A). There were four datasets generated for the HCV system, including 470 inhibitors and 1410 decoys for hepatitis C virus serine protease (NS3/4A), 19 inhibitors and 57 decoys for nonstructural protein 4B (NS4B), 40 inhibitors and 120 decoys for nonstructural protein 5A (NS5A), and 902 inhibitors and 2706 decoys for hepatitis C Virus NS5B (NS5B). Each of the 15 datasets described above was divided into a training set and a test set.

The ideal training set used to create predictive classification models that could be used to identify potent molecules from a massive database of structurally diverse compounds is diverse and large. Before modeling implementation, the Tanimoto similarity index, which is a generally used measure for evaluating molecular similarity among numerous compounds, was used to evaluate the fingerprint-based similarity using the ECFP4 fingerprint [30,31]. In general, more dissimilar compounds tend to have a smaller Tanimoto similarity index. The Tanimoto similarity indexes for a set of compounds in the training and test sets for each of 15 datasets were computed, and the results are shown in Table 1. For HIV-1, the Tanimoto similarity indexes of the compounds in the training and test sets from the 11 datasets ranged from 0.118 to 0.202 and 0.117 to 0.215, respectively. For HCV, the training sets and test sets from the four datasets had similar Tanimoto similarity indexes that ranged from 0.141 to 0.192. Table 1 shows that the compounds in the training and test sets from the 15 datasets were involved in diverse chemotypes. The dissimilarity and diversity of the training/test sets play a critical role in the performance of the classifiers.

**Figure 3:**
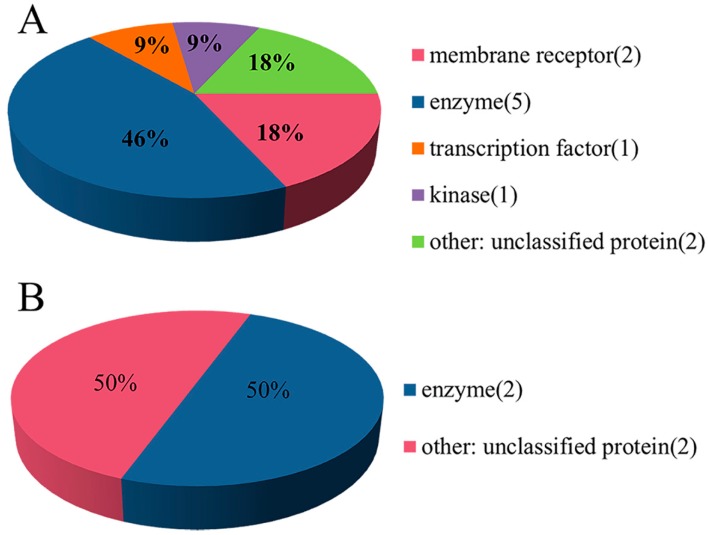
Target classification of the HIV-1 (**A**) and HCV (**B**) data sets.

In addition, according to the protein target classification rules in the ChEMBL database, 11 targets related to HIV-1 and four targets related to HCV were spatially distributed into five and two categories, respectively. As shown in Figure 3A, the HIV-1 target space (*n* = 11) consisted of five categories, including membrane receptor (*n* = 2), enzyme (*n* = 5), transcription factor (*n* = 1), kinase (*n* = 1) and unclassified protein (*n* = 2). Similarly, the HCV target space (*n* = 4) was divided into two categories, including enzyme (*n* = 2) and unclassified protein (*n* = 2) in Figure 3B. Thus, the two datasets for HIV-1 and HCV had diverse target coverage.

### 2.2. Performance Evaluation and the Comparison of the Models

Machine learning algorithms have been successfully applied to drug discovery [32]. Naïve Bayes (NB) is one of the most popular classifiers and uses the hypothesis of attribution conditional independence [23]. The support vector machine (SVM) was developed based on Vapnik’s structural risk minimization (SRM) principle [33] and shows excellent classification performance with a reduced risk of overfitting. The construction of all the classification models in this study was primarily based on the use of two classifiers (NB and SVM) and two types of fingerprints (MACCS and ECFP6). Afterwards, internal five-fold cross-validation and external test set validation were conducted.

The performance of the multiple QSAR models as determined by five-fold cross-validation is shown in Table 2. Among the 44 models of HIV-1, 86.36% models (38 out of 44) had an area under the ROC curve (AUC) value exceeding 0.9, and 79.55% of the models (35 out of 44) had a non-error rate (NER) value exceeding 0.8. In addition, the AUC values ranged from 0.834 to 1, with a median of 0.983, and the NER value ranged from 0.737 to 1, with a median of 0.946. The sixteen models of HCV had AUC and NER values exceeding 0.9 and 0.8, respectively. In addition, the AUC value ranged from 0.924 to 1, with a median of 1, and the NER value ranged from 0.801 to 1, with a median of 0.990. Thus, these results suggest that the multiple QSAR models for both HIV-1 and HCV had a remarkable capability for identifying inhibitors with low false positive rates. The detailed performance data for the training sets is given in Table S1 in the Supporting Data. Similarly, for the models of HIV-1, 75% of the models (33 out of 44) had sensitivity (SE) values exceeding 0.8, with a median of 0.918, and 93.18% of the models (41 out of 44) had specificity (SP) values exceeding 0.8, with a median of 0.997. For the models of HCV, 87.5% of the models (14 out of 16) had SE values exceeding 0.8, with a median of 0.988, and 93.8% of the models (15 out of 16) had SP values exceeding 0.8, with a median of 1.

To assess the generality of the multiple QSAR models in terms of distinguishing inhibitors from decoys, external test sets were used to perform a more rigorous validation of the training models. The assessment results for the test sets are listed in Table 3. For the 44 models of HIV-1, the AUC value ranged from 0.833 to 1, with a median value of 0.997, and the NER value ranged from 0.745 to 1, with a median value of 0.954. For the 16 models of HCV, the AUC value ranged from 0.842 to 1, with a median value of 0.994, and the NER value ranged from 0.788 to 1, with a median value of 0.959. The detailed performance data obtained for the test set validation is presented in Table S2 in the Supporting Data. The median values of SE and SP for the 11 datasets obtained for HIV-1 are 0.938 and 0.999, respectively. The median values of SE and SP for the four datasets obtained for HCV are 0.963 and 1, respectively. Moreover, the prediction accuracy (Q) is an important index for evaluating the predictive capability of models. Among the models of HIV-1 and HCV, 93.18% and 93.75% of the models, respectively, had an accuracy higher than 0.8. According to the results of the test sets, the comparable performance of the multiple QSAR models for the training and test sets indicated that the models showed no obvious overfitting. Thus, the 60 multiple QSAR models had a substantial capability to distinguish inhibitors from decoys with comparable yields and low false positive rates.

**Figure 4:**
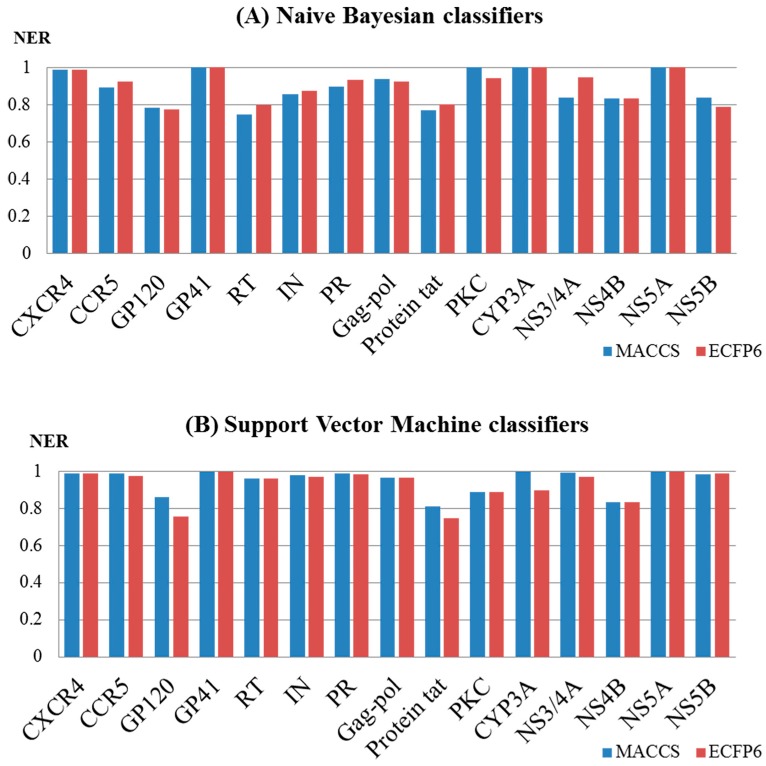
Performance of 60 multiple QSAR models built based on Naïve Bayes (**A**) and the support vector machine (**B**) using different fingerprints determined by test set validation.

To investigate the influence of different types of molecular fingerprints (MACCS and ECFP6) on the performance of the Naïve Bayes and support vector machine models, the corresponding NER values for the test sets were compared. As shown in Figure 4A,B, the NER values obtained with the MACCS and ECFP6 fingerprints based on a single machine learning method (support vector machine or Naïve Bayes) were approximately equivalent. For the models built by the support vector machine, the NER values for MACCS ranged from 0.810 to 1 with a median of 0.984, and those for ECFP6 ranged from 0.749 to 1 with a median of 0.971. For the models built according to Naïve Bayes, the NER values for MACCS ranged from 0.745 to 1 with a median of 0.891, and those for ECFP6 ranged from 0.775 to 1 with a median of 0.926. It should be noted that different fingerprints obtained different structural representations of a chemical structure, and multiple QSAR models based on different fingerprints could have an impact on the performance in terms of yield.

**Figure 5:**
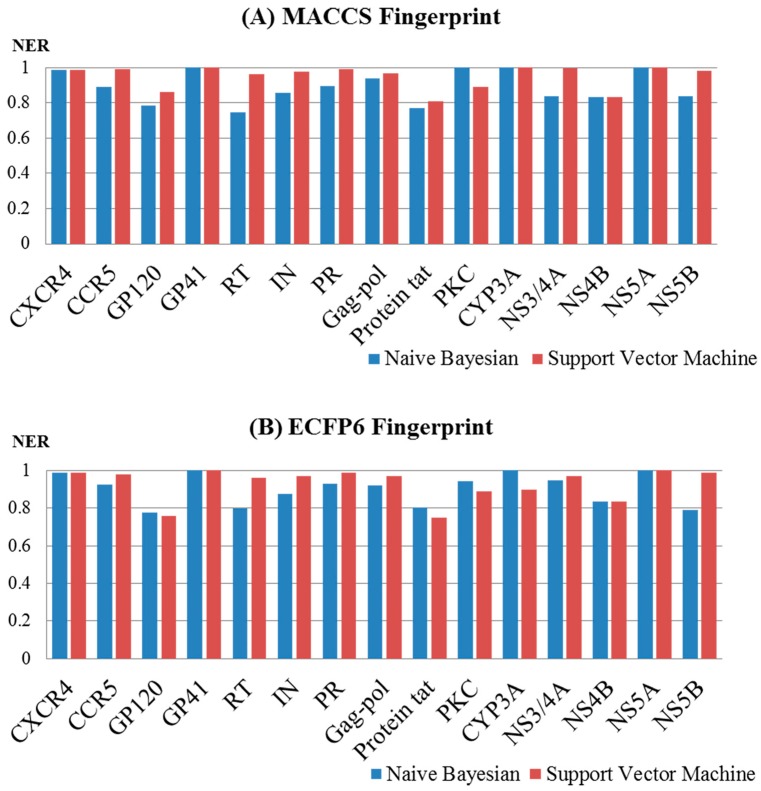
Performance of the 60 multiple QSAR models built based on MACCS (**A**) and ECFP6 (**B**) using different algorithms determined by test set validation.

Moreover, the performance of the different algorithms (NB and SVM) was also evaluated. For the MACCS fingerprint (Figure 5A), the performance of the SVM models was generally superior to that of the NB models. For example, for the models of HIV-1, the NER values for the SVM models ranged from 0.81 to 1 with a median of 0.979, and this was superior to that of the NB models, which ranged from 0.745 to 1 with a median of 0.897. For the models of HCV, the NER values for the SVM models ranged from 0.833 to 1 with a median of 0.990, and this was superior to that of the NB models, which ranged from 0.833 to 1 with a median value of 0.837. For the ECFP6 fingerprint (Figure 5B), the SVM models broadly outperformed the NB models. For the models of HIV-1, the NER values for the SVM models ranged from 0.749 to 1 with a median of 0.969, and this was superior to that of the NB models, which ranged from 0.775 to 1 with a median of 0.926. For the HCV models, the NER values for the SVM models ranged from 0.833 to 1 with a median of 0.980, and these were different from those obtained for the NB models, which ranged from 0.788 to 1 with a median value of 0.890.

In this study, on the basis of two machine learning algorithms (support vector machine and Naïve Bayes) and two fingerprints (MACC and ECFP6), four classifiers (NB_MACCS, NB_ECFP6, SVM_MACCS, SVM_ECFP6) were built for each target. As described above, for the models built by the same machine learning algorithm, the performances of the models that used MACCS were approximately equivalent to those that used ECFP6. When using the same fingerprint, the performance of the models built by the support vector machine was better than those of models built by Naïve Bayes. However, it is theoretically difficult to determine which fingerprint or algorithm is better because each algorithm or fingerprint has its own advantages and disadvantages. For example, the models built by different fingerprints or algorithms may make opposite predictions for the same molecule. Therefore, combining the results of a single classifier to predict CPIs is intelligent. One compound was predicted as positive when at least two of the four single classifiers predicted it to be active, and the corresponding CPIs were defined as potential interactions.

### 2.3. Case 1: Prediction and Analysis of the Polypharmacology of Known HIV-1 and HCV Drugs

**Figure 6:**
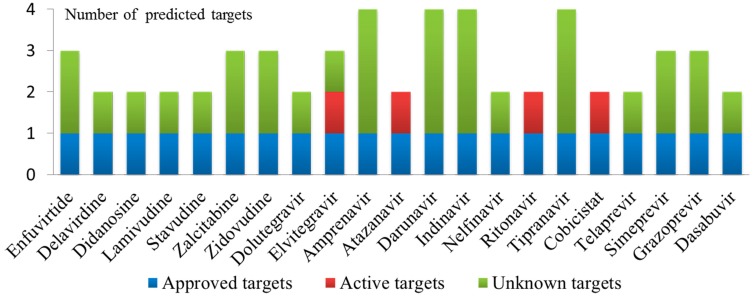
Number of predicted targets and known targets of 17 approved HIV-1 drugs and four approved HCV drugs (Approved targets: predicted targets are approved drug targets for drugs that are their inhibitor; Active targets: predicted targets for drugs with validated bioactivity; Unknown targets: predicted targets for drugs without validated bioactivity).

To explore the multiple bioactivities of known drugs, 60 multiple QSAR models based on 15 key targets were used to predict potential targets for 27 approved HIV-1 drugs and 10 approved HCV drugs. If the drug was predicted to be active by at least two of the four classifiers for each target, then the relevant CPI was defined as a potential interaction. The detailed prediction results of the polypharmacology of the known drugs are presented in Table S3 in the Supporting Data. The results show that 17 approved HIV-1 drugs and four approved HCV drugs were predicted to interact with more than one target, of which 7 approved HIV-1 drugs and four approved HCV drugs were predicted to interact with HIV-1 and HCV targets simultaneously. To verify the prediction results, the predicted targets for each drug were validated using the PubChem BioAssay database [34] and the ChEMBL database, and the corresponding binding values are presented in Table 4. Figure 6 shows a histogram of the number of predicted targets and established targets for 17 approved HIV-1 drugs and four approved HCV drugs. For example, elvitegravir, which was predicted to be active against HIV-1 RT, HIV-1 IN and HCV NS5B, could inhibit HIV-1 RT (active target) and IN (approved target) with IC_50_ values of 91 μM and 28 nM, respectively. Tipranavir, which was predicted to be active against HIV-1 GP120, HIV-1 RT, HIV-1 PR and HCV NS5B, could inhibit HIV-1 PR (approved target) with an IC_50_ value of 0.03 μM. Telaprevir, which was predicted to be active against HIV-1 PR and HCV NS3/4A, could inhibit HCV NS3/4A (approved target) with an IC_50_ value of 0.2 μM.

**Figure 7:**
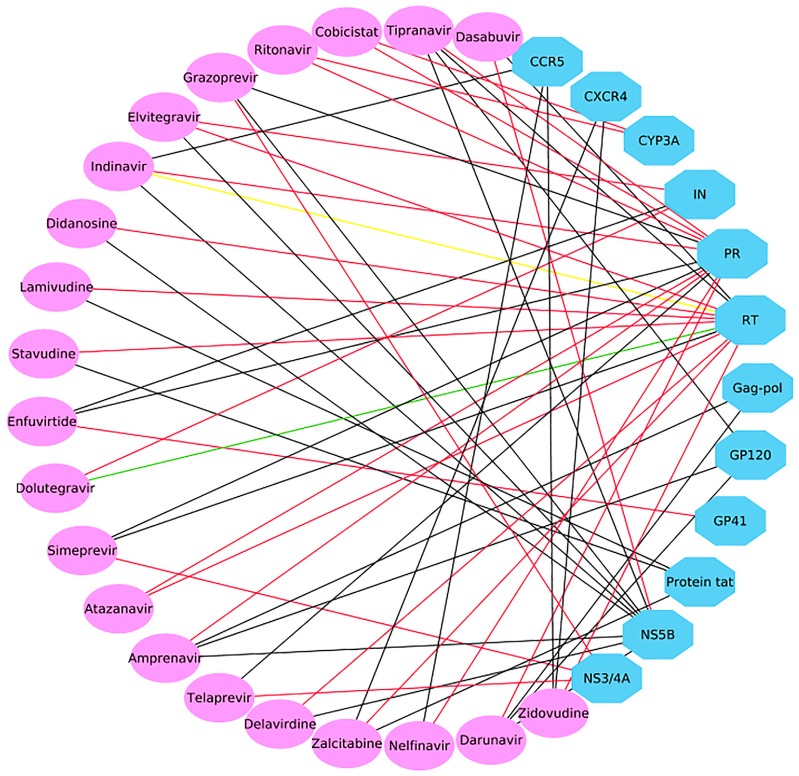
Polypharmacological analysis of 17 approved HIV-1 drugs and four approved HCV drugs based on the 60 multiple QSAR models generated in this study. Ellipses and octagons represent drug nodes and protein nodes, respectively. A red line indicates that the chemical–protein interaction (CPI) was experimentally verified as active. A black line indicates that the interaction was not proven to be worthy of further experimental validation. A green or yellow line indicates that the interaction was experimentally validated as inactive or inconclusive, respectively.

In addition, 56 CPIs were determined, as shown in Table S4 in the Supporting Data. Among them, 25 out of 56 predicted CPIs have been verified in the literature [35,36,37,38,39,40,41,42,43,44,45,46,47,48,49]; two out of 56 CPIs were predicted to be active but were validated as “inconclusive” and “inactive” [50,51]. A success rate of 44.6% (25/56) and a failure rate of 1.8% (1/56) illustrate the reliability of the multiple QSAR method. A polypharmacological interaction network of 21 drugs and predicted targets (56 CPIs) was constructed with Cytoscape 3.6 [52] (Figure 7). Figure 7 shows that each drug was predicted to have potential activity against two or three targets, which is in line with concept that “one drug can hit multiple targets”. Subsequently, the predicted CPI network identified many potential drug-target interactions that have yet to be explored, and these unverified CPIs are worthy of further experimental validation. The predicted targets may serve as new targets for approved drugs and provide promise as possible treatments for HIV/HCV coinfection.

### 2.4. Case 2: Target prediction and Analysis of Known Inhibitors

**Figure 8:**
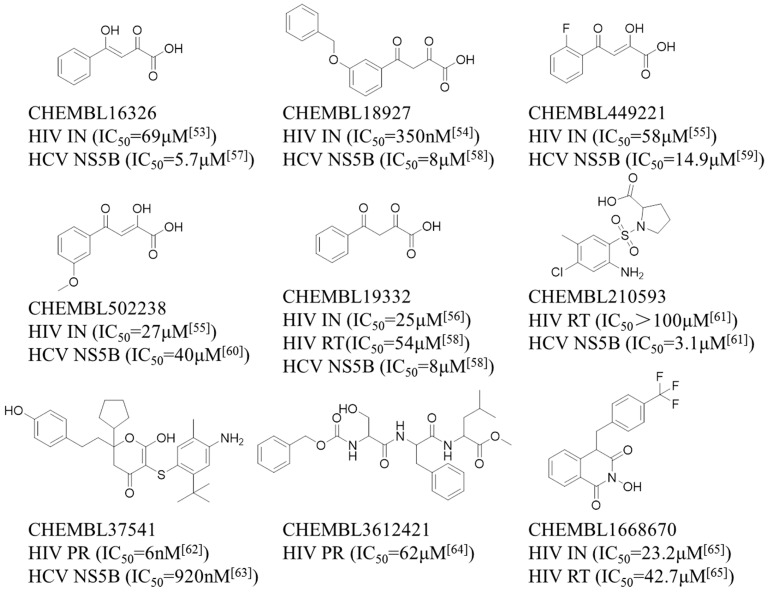
The structure and bioactivity data of the nine selected compounds.

A set of nine compounds (Figure 8) with known activity targeting at least one of the four targets (HIV-1 PR, RT, IN and HCV NS5B) that was not involved in the generation of the multiple QSAR models was retrieved from the ChEMBL database. To evaluate whether the new models could correctly differentiate between these active compounds, the classifiers built in this study were used to predict chemical interactions with HIV-1 PR, RT and IN and HCV NS5B. According to the above rules, a potential interaction is defined as a CPI when at least two out of four single classifiers predict a compound to be positive. The specific predicted results are given in Table S5 in the Supporting Data, and the comparison of the predicted and experimental results is shown in Table 5. Moreover, molecular docking provided a more detailed inspection of the binding mode for each compound. The scoring values of the top scoring poses are available in Table S6 in the Supporting Data.

Table 5 shows that all nine compounds were predicted to act against two or three targets, and seven compounds were reported to simultaneously interact with HIV-1 and HCV targets with different binding affinities. New predictions of the inhibitory activity of CHEMBL3612421 and CHEMBL1668670 against NS5B have yet to be reported experimentally. However, CHEMBL19332, a known active HIV-1 RT inhibitor, was incorrectly predicted to be inactive toward HIV-1 RT. Thus, a total of 19 CPI pairs were predicted to be positive, of which 17 CPI pairs were experimentally confirmed to be active [53,54,55,56,57,58,59,60,61,62,63,64,65]. Therefore, the prediction success rate of 89.5% (17/19) indicates the reliability of the multiple QSAR models. It is worth mentioning that the remaining two CPI pairs associated with two compounds deserve further experimental investigation, and the predicted targets might be new targets for known inhibitors. Moreover, one CPI pair shows false negatives in the prediction result.

**Figure 9:**
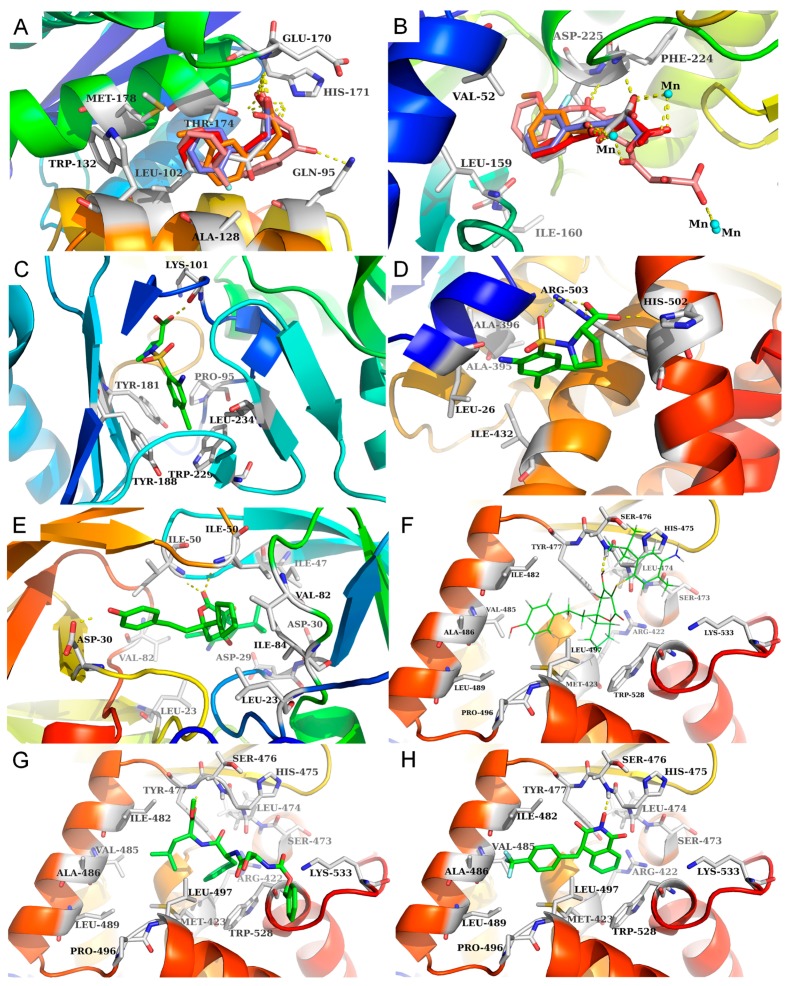
The binding modes of nine compounds in their respective binding sites. (**A**,**B**) show the binding modes of five compounds with HIV-1 IN (PDB: 4NYF) and HCV NS5B (PDB: 1GX6), respectively, including CHEMBL16326 (red), CHEMBL18927 (pink), CHEMBL449221 (purple), CHEMBL502238 (orange) and CHEMBL19332 (gray). (**C**,**D**) show the binding modes of CHEMBL210593 with HIV-1 RT (PDB: 1TL3) and HCV NS5B (PDB: 1OS5). (**E**,**F**) show the binding modes of CHEMBL37541 with HIV-1 PR (PDB: 4MC1) and HCV NS5B (PDB: 1OS5). (**G**,**H**) show the binding modes of two compounds (CHEMBL3612421 and CHEMBL1668670) with HCV NS5B (PDB 1OS5). (The cocrystallized molecules are shown as lines, whereas the molecules modeled into the receptors are shown as sticks).

The results in Table 5 show that five compounds (CHEMBL16326, CHEMBL18927, CHEMBL449221, CHEMBL502238 and CHEMBL19332) were predicted to be potentially active against HIV-1 IN and HCV NS5B, and all of these were also experimentally confirmed to be HIV-1 IN and HCV NS5B inhibitors in the literature [53,54,55,56,57,58,59,60]. In particular, five compounds with analogous structures have similar protein-ligand interactions with the active regions of HIV-1 IN and HCV NS5B. As shown in Figure 9, the hydrophobic groups in those five compounds, such as the benzene ring in CHEMBL16326 and the monofluorobenzene ring in CHEMBL449221, are located in hydrophobic pockets formed by Leu102, Ala128, Trp132, Thr174 and Met178 of HIV-1 IN (see Figure 9A) and Val52, Leu159 and Ile160 of HCV NS5B (see Figure 9B). The diketo acid moieties of these five compounds form hydrogen bonds with Glu170 and His171 in the active site of HIV-1 IN (see Figure 9A) and with Phe224 and Asp225 and via Mn2+ ions (metal chelation) in the catalytic activity site of HCV NS5B (see Figure 9B). Thus, HIV-1 IN and HCV NS5B can be affected by diketo acid inhibitors, which disturb enzymatic function during the process of virus replication.

CHEMBL210593 was predicted to be a HIV-1 RT and HCV NS5B inhibitor with an IC_50_ > 100 μM and 3.1 μM, respectivelyn [61]. Figure 9 shows that the 3-chloro-p-methylaniline in CHEMBL210593 occupies a hydrophobic pocket formed by Tyr181, Tyr188 and Trp229 in HIV-1 RT (see Figure 9C) and Leu26, Ala395, Ala396 and Ile432 in HCV NS5B (see Figure 9D). The carboxyl group of CHEMBL210593 forms hydrogen bonds with the main chain carbonyl oxygen of Lys101 in HIV-1 RT (see Figure 9C) and the side chains of Arg503 and His502 in HCV NS5B (see Figure 9D). Therefore, proline sulfonamide inhibitors may affect HIV-1 RT and HCV NS5B and influence their enzymatic function.

CHEMBL37541 and CHEMBL3612421 were also predicted to be active against HIV-1 PR and HCV NS5B. CHEMBL37541 is a known HIV-1 PR and HCV NS5B inhibitor with IC_50_ values of 6 nM and 920 nM, respectively [62,63]. CHEMBL3612421 is a specific HIV-1 PR inhibitor with an IC_50_ of 62 μM [64], but inhibitory activity against NS5B by CHEMBL3612421 has not yet been reported. In CHEMBL37541, the dihydropyrone moiety forms hydrogen bonds directly with Ile50 in HIV-1 PR (see Figure 9E) and Ser476 in HCV NS5B (see Figure 9F). The 4-ethylphenol and cyclopentane groups fit into hydrophobic pockets consisting of the residues Leu23, Leu23′, Val82, Val82′, Ile84 and Ile84′ in HIV-1 PR (see Figure 9E) and the residues Met423, Ile482, Val485, Ala486, Leu489, Leu497 and Trp528 in HCV NS5B (see Figure 9F). A newly predicted CPI pair involving CHEMBL3612421 and HCV NS5B is presented in Figure 9G. CHEMBL3612421 forms a hydrogen bond with Tyr477 in the thumb II region of HCV NS5B, and its benzene ring and dimethyl group are located in a hydrophobic region consisting of the residues Met423, Ile482, Val485, Ala486, Leu489, Leu497 and Trp528. Similarly, dihydropyrone inhibitors can affect HIV-1 PR and HCV NS5B and influence enzymatic function.

CHEMBL1668670 was also predicted to be an HIV-1 RT, IN and HCV NS5B inhibitor, and it has demonstrated bioactivity only against HIV-1 RT (IC_50_ = 42.7 μM) and IN (IC_50_ = 23.2 μM) [65]. There have been no reports on the inhibitory activity of CHEMBL1668670 against NS5B. A newly predicted CPI pair involving CHEMBL1668670 and NS5B is shown in Figure 9H. CHEMBL1668670 forms a hydrogen bond with Ser476 in the thumb II region of HCV NS5B, and its trifluorobenzene ring interacts with a hydrophobic region consisting of the residues Met423, Ile482, Val485, Ala486, Leu489 and Leu497.

Thus, the results show that it is possible to design specific inhibitors targeting HIV-1 targets and HCV targets simultaneously. In addition, an individual compound was determined to have coinhibitory ability against both virus targets if it was able to satisfy the requirements for binding the HIV/HCV target active sites in terms of the molecular shape, size and physical–chemical properties. In short, multiple QSAR models are expected to contribute to the design and identification of different types of co-inhibitors with excellent affinities that selectively and simultaneously target multiple HIV-1 and HCV targets for the treatment of HIV/HCV coinfection.

## 3. Discussion

In this study, based on the multiple QSAR method, 44 binary classifiers for 11 targets related to HIV-1 and 16 binary classifiers for four targets related to HCV were established to predict the CPIs. In addition, the predictive reliability of the models was confirmed by five-fold cross-validation and test set validation.

To illustrate the application of the models, two different cases were investigated to systematically predict the multiple bioactivities of 27 approved HIV-1 drugs and 10 approved HCV drugs (case 1) and nine compounds that were known to be active against at least one target of HIV-1 and HCV (case 2). For case 1, 21 approved drugs were predicted to be active against more than one biological target. The predictions were confirmed on the basis of reported bioactivity data with a success rate of 44.6% and a failure rate of 1.8%. For case 2, nine active compounds against at least one target (HIV-1 PR, RT, IN or HCV NS5B) were predicted to be multitarget inhibitors by multiple QSAR models with a success rate of 89.5%. The prediction results demonstrate that the machine learning models had high specific and low false negative rates in this study. Two possible explanations for the occurrence of false negatives are as follows. First, the molecular fingerprint was unable to sufficiently represent the molecular structural information. Second, the inherent limitations of machine learning and the composition of the training set had a significant impact on the accuracy of the models. In short, the predictive results of the above two cases indicate that multiple QSAR models showed high performance in the prediction of multitarget inhibitors with activity against both HIV and HCV.

## 4. Conclusions

In the past, “one gene, one drug, one disease” was the main paradigm of drug discovery. However, with the introduction of the concept of polypharmacology, multitarget therapy using a single drug that simultaneously inhibits two or more targets may provide a new strategy. Here, multiple QSAR models were used to identify compounds acting on multiple targets for the treatment of HIV/HCV coinfection.

As mentioned above, the multiple QSAR method achieved satisfactory prediction accuracy, and the machine learning models were applicable to potential target identification in this study. The limited chemical diversity of the training set makes the QSAR models suitable for predicting the knowledge or information that served as the basis on which the models were developed in the chemical space. Thus, it is unreasonable to predict a whole chemical space using a single QSAR model. A prediction based on QSAR is significant only if the specific compound being predicted falls within the HIV/HCV coinfection basis of the model. In addition, QSAR modeling neglects the interactions between receptors and ligands, unlike molecular docking. Therefore, it is necessary to choose a reasonable single method or combination of methods according to the specific situation.

In summary, the computational methods utilized in this study could effectively detect multitargeted co-inhibitors for the suppression of HIV/HCV coinfection and have potential applications for the identification of new targets, the virtual screening of multitarget drugs and drug repurposing for the treatment of other diseases. In addition, we hope that our research will be helpful in the clinical treatment of HIV/HCV coinfection.

## 5. Materials and Methods

### 5.1. Dataset Preparation

The Therapeutic Target Database (TTD) [28] and ChEMBL Database (version 23, https://www.ebi.ac.uk/chembl/) were used to determine the clinical targets of anti-HIV and anti-HCV therapy. We used keywords with ‘HIV-1′, ‘Human Immunodeficiency Virus type-1′, ‘HCV’ and ‘Hepatitis C Virus’ as the retrieval conditions, and the search results were further filtered to identify drug candidates that had at minimum entered phase I clinical trials.

In this study, the known small molecule inhibitors of the HIV-1 or HCV targets were downloaded from the ChEMBL Database. The datasets were processed according to the following requirements: (1) the compound, with an IC_50_, EC_50_ or K_i_ ≤ 20 μM, was retained as positive (expressed as ‘+1′); (2) salts were converted into the homologous acid or base and water molecules in hydrates were eliminated; (3) duplicate molecules were deleted. DecoyFinder software [66,67] was applied to prepare a set of decoy compounds (three decoys for each known inhibitor) [21] by using information from the ZINC database (version 12, http://zinc.docking.org/), and the decoy compounds were denoted as negative (expressed as ‘−1’) for each target. Specifically, the decoy compounds were selected based on the following constraints: (i) the MW was within 25 Da of that of the corresponding positive compound; (ii) the number of RB, HBDs, HBAs were ±1, ±1 and ±2 of those of the positive compound, respectively; (iii) the LogP was ±1 of that of the positive compound; (iv) the Tanimoto coefficient of a potential decoy vs that of the active compound and that of a potential decoy vs a previously selected decoy were, by default, 0.75 and 0.9, respectively [66]. Finally, the dataset including inhibitors and decoys for each target was divided into a training set (70%) and a test set (30%) using the default values of the parameters of the partitioning node in KNIME (version 3.4.2, https://www.knime.com) [68]. The above data processing procedures were performed in KNIME, an open-source data analysis and cheminformatic software.

### 5.2. Molecular Representation

Each compound in the above data sets was represented using two different types of binary fingerprint descriptors (MACCS and ECFP6) as chemical descriptors calculated in KNIME. MACCS is a common 2D molecular fingerprint descriptor composed of 166 structural keys. Most of the important chemical features are covered despite the small length [69]. Binary fingerprints (FPs) completely characterize all of the structural fragments in the molecule in a binary form [70]. It is different from classical descriptors, as fingerprints encode molecular structural information as a range of binary bits and can only be deciphered used as a whole. Additionally, extended-connectivity fingerprints (ECFPs), also known as “circular fingerprints”, are state-of-the-art topological fingerprints [27]. In this study, the ECFPs were generated with the default parameters of 1024 bits, 2 bits per pattern and a radius of 3 bonds, which was equivalent to ECFP6, using Fingerprint node [71,72] in KNIME. To date, MACCS and ECFPs have been widely used in drug discovery on the basis of their characteristics of rapid calculation and their representation of many different molecular features and stereochemical information.

### 5.3. Multiple QSAR Models Generation

In this study, all classification models were built by combining two machine learning algorithms (Naïve Bayes and support vector machine) and two molecular fingerprints (MACCS and ECFP6). Therefore, four classifiers (NB_MACCS, NB_ECFP6, SVM_MACCS and SVM_ECFP6) were generated for each target. The number of multiple QSAR models generated for the 11 HIV-1 targets and four HCV targets were 44 and 16, respectively.

#### 5.3.1. Naïve Bayes (NB)

The Naïve Bayes classification models were built using KNIME (version 3.4.2). Bayesian classification is widely used to solve classification problems based on Bayesian theory, such as the discrimination between active and inactive compounds, by building a classifier. Naïve Bayes is based on the occurrence frequency of various descriptors found in two or more groups of molecules and is used to distinguish optimally between these datasets. The details of the Bayesian theorem are given in Equations (1)–(3):

(1) $$P(y_{i}|x)=\frac{P(x|y_{i})P\left(y_{i}\right)}{P\left(x\right)}$$

(2) $$P\left(y_{i}|x\right)=\frac{P\left(y_{i}\right)}{P\left(x_{1},x_{2},\cdots ,x_{m}\right)}P\left(x_{1},x_{2},\cdots ,x_{m}{|y}_{i}\right)$$

(3) $$P(y_{i}|x)=\frac{P\left(y_{i}\right)}{P\left(x_{1},x_{2},\cdots ,x_{m}\right)}\prod_{j=1}^{d}P(x_{j}|y_{i})$$

In general, x represents molecular descriptors such as molecular weight, logP or molecular fingerprint, y represents whether a compound will exhibit a desired biological activity such as enzyme inhibition, P(y_i_) is the prior probability of y_i_ being true without any knowledge of x, and P(x|y_i_) is the conditional probability of y_i_ being true given x. P(x_1_,x_2_, …, x_m_) represents the marginal probabilities of the molecular descriptors that will appear in the training set. Because of the assumption of attribution independence, Equation (2) is simplified to Equation (3), which is also known as the Naïve Bayes formula.

#### 5.3.2. Support Vector Machine (SVM)

Based on the principle of structural risk minimization (SRM) [73], SVM has been developed as a useful classification technique. The main idea is to convert input sample space into a high-dimensional space, in which a hyperplane with a maximal margin is generated to describe a nonlinear classification boundary. Importantly, the classification is achieved through a kernel function. There are four basic kernels: linear, polynomial, radial basis function (RBF) and sigmoid [30]. In most cases, RBF is preferred [74]. In this study, the RBF kernel function was used. The penalty parameter C and kernel parameter γ that were used in the SVM were optimized through five-fold cross-validation in SVM, and the final optimal values of C and γ that provided the best accuracy were selected for generating the final classifiers [75].

### 5.4. Performance Evaluation of the Multiple QSAR Models

All models were evaluated by internal five-fold cross-validation and external test set validation. In five-fold cross-validation, the training set was divided into five subsets equally. Four subsets were used to train the model, and the remaining subset was used as an internal validation set.

The major evaluation indexes included true positives (TP), true negatives (TN), false positives (FP), false negatives (FN), sensitivity (SE), specificity (SP), the overall prediction accuracy (Q) and the receiver operating characteristic curve (ROC). Among these, ROC curves are often used to estimate the merits of a binary classifier, in which the false positive rate (FPR) and true positive rate (TPR) are used as the abscissa and the ordinate, respectively. Generally, the AUC value ranges from 0 to 1, and the higher the value, the better the classification capability of the model is.

(4) $$\mathrm{NER}=\frac{\sum_{g=1}^{G}{\mathrm{Sn}}_{g}}{G}$$

In addition, the average value of the sensitivity for all classes, also called the non-error rate (NER), was calculated according to Equation (4). The value of NER ranges from 0 to 1, and the value of NER is 1, indicating a perfect classification [76].

### 5.5. Molecular Docking

The docking algorithm Glide [77] was used for molecular docking and the analysis of the binding modes between the small molecules and target proteins. The crystal structures of HIV-1 IN (PDB: 4NYF) [78], HIV-1 RT (PDB: 1TL3) [79], HIV-1 PR (PDB: 4MC1) [80] and HCV NS5B (PDB: 1OS5 and 1GX6) [81,82] were prepared and used to build the receptor grid. The grid was centered on the ligand in the co-crystallized complex or the key residues of the proteins, and then the Glide SP protocol was utilized for docking. For grid generation and docking, all of the parameters used were the default values.

## Figures and Tables

**Table 1:** Detailed statistical analysis of the 15 datasets.

Object	Target	Training Set				Test Set			
		Inhibitors	Decoys	Total	Tanimoto Similarity Index	Inhibitors	Decoys	Total	Tanimoto Similarity Index
HIV-1	CXCR4	113	339	452	0.152	44	132	176	0.158
	CCR5	255	766	1021	0.161	115	344	459	0.160
	GP120	208	624	832	0.142	85	255	340	0.147
	GP41	14	42	56	0.172	7	21	28	0.189
	RT	2279	6837	9116	0.124	977	2931	3908	0.124
	IN	1617	4850	6467	0.134	598	1795	2393	0.135
	PR	2837	8509	11,346	0.141	1274	3824	5098	0.141
	Gag-pol	69	205	274	0.147	32	98	130	0.146
	Protein tat	302	906	1208	0.118	136	408	544	0.117
	PKC	20	58	78	0.162	9	29	38	0.169
	CYP3A	10	30	40	0.202	5	15	20	0.215
HCV	NS5B	649	1948	2597	0.141	253	758	1011	0.141
	NS4B	13	37	50	0.178	6	20	26	0.192
	NS3/4A	334	1000	1334	0.145	136	410	546	0.145
	NS5A	27	80	107	0.182	13	40	53	0.182

**Table 2:** Performance results for the 60 multiple QSAR models determined by five-fold cross-validation.

Object	Target	MACCS				ECFP6			
		NB		SVM		NB		SVM	
		AUC	NER	AUC	NER	AUC	NER	AUC	NER
HIV-1	CXCR4	0.992	0.962	0.998	0.978	0.994	0.969	0.997	0.973
	CCR5	0.977	0.878	0.997	0.990	0.986	0.936	0.996	0.976
	GP120	0.845	0.799	0.926	0.851	0.880	0.775	0.923	0.737
	GP41	0.941	0.964	0.958	0.964	0.963	0.964	0.934	0.786
	RT	0.838	0.763	0.994	0.960	0.898	0.797	0.994	0.955
	IN	0.930	0.863	0.996	0.976	0.949	0.878	0.994	0.960
	PR	0.980	0.908	0.999	0.987	0.982	0.925	0.997	0.987
	Gag-pol	0.966	0.942	0.984	0.949	0.967	0.925	0.980	0.935
	Protein tat	0.834	0.745	0.909	0.792	0.870	0.805	0.901	0.739
	PKC	0.991	0.991	1	0.975	0.994	0.925	0.988	0.925
	CYP3A	1	1	1	1	1	1	1	0.950
HCV	NS3/4A	0.990	0.853	1	0.993	0.994	0.953	1	0.987
	NS4B	1	1	1	1	1	1	1	0.885
	NS5A	1	1	1	1	1	1	1	1
	NS5B	0.924	0.863	1	0.987	0.946	0.801	1	0.984

**Table 3:** The test results for the performance of the 60 multiple QSAR models determined by test set validation.

Object	Target	MACCS				ECFP6			
		NB		SVM		NB		SVM	
		AUC	NER	AUC	NER	AUC	NER	AUC	NER
HIV-1	CXCR4	1	0.989	1	0.989	1	0.989	1	0.989
	CCR5	0.981	0.891	0.999	0.990	0.995	0.926	1	0.978
	GP120	0.881	0.784	0.940	0.861	0.883	0.775	0.910	0.759
	GP41	1	1	1	1	1	1	1	1
	RT	0.837	0.745	0.995	0.964	0.899	0.799	0.994	0.963
	IN	0.934	0.856	0.997	0.979	0.947	0.876	0.996	0.972
	PR	0.975	0.897	1	0.991	0.982	0.932	0.999	0.987
	Gag-pol	0.977	0.938	0.988	0.969	0.975	0.923	1	0.969
	Protein tat	0.833	0.771	0.920	0.810	0.864	0.803	0.926	0.749
	PKC	1	1	1	0.889	1	0.944	1	0.889
	CYP3A	1	1	1	1	1	1	1	0.900
HCV	NS3/4A	0.986	0.836	1	0.996	0.987	0.946	1	0.971
	NS4B	0.842	0.833	0.925	0.833	0.867	0.833	0.908	0.833
	NS5A	1	1	1	1	1	1	1	1
	NS5B	0.907	0.837	1	0.984	0.944	0.788	1	0.988

**Table 4:** Known interactions among the CPIs predicted by the multiple QSAR models for approved drugs.

Drug Name	Predicted Target	Known Activity	Reference
Enfuvirtide	GP41	IC_50_ = 90.2 nM	[35]
Delavirdine	RT	IC_50_ = 0.17 μM	[36]
Didanosine	RT	IC_50_ = 4.6 μM	[42]
Lamivudine	RT	IC_50_ = 0.60 μM	[42]
Stavudine	RT	IC_50_ = 1.9 μM	[42]
Zalcitabine	RT	IC_50_ = 0.13 μM	[42]
Zidovudine	RT	IC_50_ = 0.13 μM	[42]
Dolutegravir	RT	Inactive	[50]
Dolutegravir	IN	IC_50_ = 2.7 nM	[43]
Elvitegravir	RT	IC_50_ = 91 μM	[44]
Elvitegravir	IN	IC_50_ = 28 nM	[44]
Amprenavir	PR	IC_50_ = 0.15 μM	[45]
Atazanavir	RT	IC_50_ = 26 nM	[46]
Atazanavir	PR	IC_50_ = 3.5 nM	[47]
Darunavir	PR	IC_50_ = 3.0 nM	[47]
Indinavir	RT	Inconclusive	[51]
Indinavir	PR	IC_50_ < 0.01 μM	[51]
Nelfinavir	PR	IC_50_ = 0.53 nM	[48]
Ritonavir	PR	IC_50_ = 0.6 nM	[49]
Ritonavir	CYP3A	IC_50_ = 0.11 μM	[49]
Tipranavir	PR	IC_50_ = 0.03 μM	[37]
Cobicistat	PR	IC_50_ = 0.15 μM	[49]
Cobicistat	CYP3A	IC_50_ > 30 μM	[49]
Telaprevir	NS3/4A	IC_50_ = 0.2 μM	[38]
Simeprevir	NS3/4A	IC_50_ = 10 nM	[39]
Grazoprevir	NS3/4A	IC_50_ = 0.07 nM	[40]
Dasabuvir	NS5B	IC_50_ = 0.6 μM	[41]

**Table 5:** Predicted and experimental activity of the nine selected compounds against HIV-1 protease (PR), reverse transcriptase (RT), integrase (IN) and HCV NS5B.

CHEMBL ID	PR/Pred ^1^	PR/Exp ^2^	IN/Pred ^3^	IN/Exp ^4^	RT/Pred ^5^	RT/Exp ^6^	NS5B/Pred ^7^	NS5B/Exp ^8^
CHEMBL16326	false	— ^9^	true	active	false	—	true	active
CHEMBL18927	false	—	true	active	false	—	true	active
CHEMBL449221	false	—	true	active	false	—	true	active
CHEMBL502238	false	—	true	active	false	—	true	active
CHEMBL19332	false	—	true	active	false	active	true	active
CHEMBL210593	false	—	false	—	true	active	true	active
CHEMBL37541	true	active	false	—	false	—	true	active
CHEMBL3612421	true	active	false	—	false	—	true	—
CHEMBL1668670	false	—	true	active	true	active	true	—

^1^ “PR/Pred” shows the predicted result of a compound against PR. ^2^ “PR/Exp” shows the experimental result of a compound against PR.^3^ “IN/Pred” shows the predicted result of a compound against IN. ^4^ “IN/Exp” shows the experimental result of a compound against IN. ^5^ “RT/Pred” shows the predicted result of a compound against RT. ^6^ “RT/Exp” shows the experimental result of a compound against RT. ^7^ “NS5B/Pred” shows the predicted result of a compound against NS5B. ^8^ “NS5B/Exp” shows the experimental result of a compound against NS5B. ^9^ “—” indicates that the predicted result has not been verified by experiments.

## Supplementary Materials

Supplementary materials can be found at https://www.mdpi.com/1422-0067/20/14/3572/s1.
