# Supplementary material for: Targeting HIV/HCV Coinfection Using a Machine Learning-Based Multiple Quantitative Structure-Activity Relationships (Multiple QSAR) Method
Source: Int J Mol Sci. 2019 Jul 22;20(14):3572. doi: 10.3390/ijms20143572 (PMC6678913; doi:10.3390/ijms20143572)
Supplement: Supplementary file 1 [file ijms-20-03572-s001.zip › Supplementary File/C8YNVCWC-certificate.pdf]

ACS Authoring Services

EDITORIAL CERTIFICATE

This document certifies that the manuscript listed below was edited for proper English language, grammar, punctuation, spelling, and overall style by one or more of the highly qualified native English speaking editors at ACS Authoring Services.

MANUSCRIPT TITLE:

Targeting against HIV/HCV Co-infection using Machine Learning-based multiple quantitative structure-activity relationships (multiple QSAR) Methods

AUTHORS:

Yu Wei, Wei Li, Tengfei Du, Zhangyong Hong, Jianping Lin

DATE ISSUED:

June 25, 2019

CERTIFICATE VERIFICATION KEY:

DD19-DBDE-E309-FFE2-61AE

This certificate may be verified at secure.authoringservices.acs.org/certificate. This document certifies that the manuscript listed above was edited for proper English language, grammar, punctuation, spelling, and overall style by highly qualified native English speaking editors at ACS AuS. Neither the research content nor the authors' intentions were altered in any way during the editing process. Documents receiving this certification should be English-ready for publication; however, the author is able to accept or reject our suggestions and changes. To verify the final ACS AuS edited version, please visit our verification page. If you have any questions or concerns about this edited document, please contact ACS AuS at support@authoringservices.acs.org. AUTHORS: Please use caution when sharing your certificate verification code. Anyone you provide this code can download the final version of the ACS AuS manuscript. Please note that this certificate DOES NOT guarantee manuscript acceptance.

ACS Authoring Services provides editing services for researchers around the world. Our top-quality editors are all native English speakers from America's top universities. Our editors come from nearly every research field and possess the highest qualifications to edit research manuscripts written by non-native English speakers. For more information about our services, please visit authoringservices.acs.org.
