# Supplementary material for: Targeting HIV/HCV Coinfection Using a Machine Learning-Based Multiple Quantitative Structure-Activity Relationships (Multiple QSAR) Method
Source: Int J Mol Sci. 2019 Jul 22;20(14):3572. doi: 10.3390/ijms20143572 (PMC6678913; doi:10.3390/ijms20143572)
Supplement: Supplementary file 1 [file ijms-20-03572-s001.zip › Supplementary File/Supplementary materials.docx]

Targeting HIV/HCV coinfection using a machine learning-based multiple quantitative structure-activity relationships (multiple QSAR) method

Yu Wei^1,#^, Wei Li^1,3,#^, Tengfei Du^1^, Zhangyong Hong^2*^, Jianping Lin ^1,3,4*^

^1^ State Key Laboratory of Medicinal Chemical Biology, College of Pharmacy and Tianjin Key Laboratory of Molecular Drug Research, Nankai University, Haihe Education Park, 38 Tongyan Road, Tianjin 300353, China

^2^ State Key Laboratory of Medicinal Chemical Biology, College of Life Sciences, Nankai University, 94 Weijin Road, Tianjin 300071, China

^3^ Platform of Pharmaceutical Intelligence, Tianjin International Joint Academy of Biomedicine, Tianjin, 300000, China

^4^ Biodesign Center, Tianjin Institute of Industrial Biotechnology, Chinese Academy of Sciences, Tianjin 300308, China

#These authors contributed equally to this work.

* Correspondence:

Jianping Lin, State Key Laboratory of Medicinal Chemical Biology, College of Pharmacy and Tianjin Key Laboratory of Molecular Drug Research, Nankai University, Haihe Education Park, 38 Tongyan Road, Tianjin 300353, China, email: [jianpinglin@nankai.edu.cn](mailto:jianpinglin@nankai.edu.cn)

Zhangyong Hong, College of Life Sciences, Nankai University, 94 Weijin Road, Tianjin 300071, China, email: [hongzy@nankai.edu.cn](mailto:hongzy@nankai.edu.cn)

**Contents:**

**Table S1.** The detailed performance of 60 multiple QSAR models by 5-fold cross-validation using NB and SVM classifiers.

**Table S2.** The detailed performance of 60 multiple QSAR models by the test set validation using NB and SVM classifiers.

**Table S3.** The prediction results of polypharmacology for 27 approved HIV-1 drugs and 10 approved HCV drugs.

**Table S4.** 56 chemical-protein interaction pairs predicted by all classifiers.

**Table S5.** The prediction results of polypharmacology for known active compounds toward HIV-1 and HCV.

**Table S6**. The docking score of 9 selected compounds and 6 drugs that towards HIV-1 PR, RT, IN and HCV NS5B.

**Table S1. The detailed performance of 60 multiple QSAR models by 5-fold cross-validation using NB and SVM classifiers.**

| **Object** | **Target** | **Finger**  **print** | **NB** | | | | | | | **SVM** | | | | | | |
| --- | --- | --- | --- | --- | --- | --- | --- | --- | --- | --- | --- | --- | --- | --- | --- | --- |
|  |  |  | **TP^a^** | **FP^b^** | **TN^c^** | **FN^d^** | **SE^e^** | **SP^f^** | **Q^g^** | **TP** | **FP** | **TN** | **FN** | **SE** | **SP** | **Q** |
| HIV-1 | CXCR4 | MACCS | 105 | 2 | 337 | 8 | 0.929 | 0.994 | 0.978 | 108 | 0 | 339 | 5 | 0.956 | 1 | 0.989 |
|  |  | ECFP6 | 109 | 9 | 330 | 4 | 0.965 | 0.973 | 0.971 | 107 | 0 | 339 | 6 | 0.947 | 1 | 0.987 |
|  | CCR5 | MACCS | 196 | 10 | 756 | 59 | 0.769 | 0.987 | 0.932 | 251 | 4 | 762 | 4 | 0.984 | 0.995 | 0.992 |
|  |  | ECFP6 | 248 | 77 | 689 | 7 | 0.973 | 0.899 | 0.918 | 243 | 0 | 766 | 12 | 0.953 | 1 | 0.988 |
|  | GP120 | MACCS | 166 | 125 | 499 | 42 | 0.798 | 0.8 | 0.799 | 149 | 9 | 615 | 59 | 0.716 | 0.986 | 0.918 |
|  |  | ECFP6 | 183 | 206 | 418 | 25 | 0.88 | 0.67 | 0.722 | 99 | 1 | 623 | 109 | 0.476 | 0.998 | 0.868 |
|  | GP41 | MACCS | 13 | 0 | 42 | 1 | 0.929 | 1 | 0.982 | 13 | 0 | 42 | 1 | 0.929 | 1 | 0.982 |
|  |  | ECFP6 | 13 | 0 | 42 | 1 | 0.929 | 1 | 0.982 | 8 | 0 | 42 | 6 | 0.571 | 1 | 0.893 |
|  | RT | MACCS | 1493 | 876 | 5961 | 786 | 0.655 | 0.872 | 0.818 | 2103 | 13 | 6824 | 176 | 0.923 | 0.998 | 0.979 |
|  |  | ECFP6 | 1943 | 1774 | 5063 | 336 | 0.853 | 0.741 | 0.769 | 2078 | 6 | 6831 | 201 | 0.912 | 0.999 | 0.977 |
|  | IN | MACCS | 1249 | 227 | 4623 | 368 | 0.772 | 0.953 | 0.908 | 1546 | 24 | 4826 | 71 | 0.956 | 0.995 | 0.985 |
|  |  | ECFP6 | 1409 | 558 | 4292 | 208 | 0.871 | 0.885 | 0.882 | 1492 | 10 | 4840 | 125 | 0.923 | 0.998 | 0.979 |
|  | PR | MACCS | 2367 | 160 | 8349 | 470 | 0.834 | 0.981 | 0.944 | 2767 | 16 | 8493 | 70 | 0.975 | 0.998 | 0.992 |
|  |  | ECFP6 | 2741 | 996 | 7513 | 96 | 0.966 | 0.883 | 0.904 | 2765 | 2 | 8507 | 72 | 0.975 | 1 | 0.993 |
|  | Gag-pol | MACCS | 63 | 6 | 199 | 6 | 0.913 | 0.971 | 0.956 | 62 | 0 | 205 | 7 | 0.899 | 1 | 0.974 |
|  |  | ECFP6 | 64 | 16 | 189 | 5 | 0.928 | 0.922 | 0.923 | 60 | 0 | 205 | 9 | 0.87 | 1 | 0.967 |
|  | Protein tat | MACCS | 209 | 183 | 723 | 93 | 0.692 | 0.798 | 0.772 | 182 | 17 | 889 | 120 | 0.603 | 0.981 | 0.887 |
|  |  | ECFP6 | 237 | 159 | 747 | 65 | 0.785 | 0.825 | 0.815 | 146 | 5 | 901 | 156 | 0.483 | 0.994 | 0.867 |
|  | PKC | MACCS | 20 | 1 | 57 | 0 | 1 | 0.983 | 0.987 | 19 | 0 | 58 | 1 | 0.95 | 1 | 0.987 |
|  |  | ECFP6 | 17 | 0 | 58 | 3 | 0.85 | 1 | 0.962 | 17 | 0 | 58 | 3 | 0.85 | 1 | 0.962 |
|  | CYP3A | MACCS | 10 | 0 | 30 | 0 | 1 | 1 | 1 | 10 | 0 | 30 | 0 | 1 | 1 | 1 |
|  |  | ECFP6 | 10 | 0 | 30 | 0 | 1 | 1 | 1 | 9 | 0 | 30 | 1 | 0.9 | 1 | 0.975 |
| HCV | NS3/4A | MACCS | 236 | 1 | 999 | 98 | 0.707 | 0.999 | 0.926 | 329 | 0 | 1000 | 5 | 0.985 | 1 | 0.996 |
|  |  | ECFP6 | 331 | 85 | 915 | 3 | 0.991 | 0.915 | 0.934 | 325 | 0 | 1000 | 9 | 0.973 | 1 | 0.993 |
|  | NS4B | MACCS | 13 | 0 | 37 | 0 | 1 | 1 | 1 | 13 | 0 | 37 | 0 | 1 | 1 | 1 |
|  |  | ECFP6 | 13 | 0 | 37 | 0 | 1 | 1 | 1 | 10 | 0 | 37 | 3 | 0.769 | 1 | 0.94 |
|  | NS5A | MACCS | 27 | 0 | 80 | 0 | 1 | 1 | 1 | 27 | 0 | 80 | 0 | 1 | 1 | 1 |
|  |  | ECFP6 | 27 | 0 | 80 | 0 | 1 | 1 | 1 | 27 | 0 | 80 | 0 | 1 | 1 | 1 |
|  | NS5B | MACCS | 577 | 318 | 1630 | 72 | 0.889 | 0.837 | 0.85 | 633 | 2 | 1946 | 16 | 0.975 | 0.999 | 0.993 |
|  |  | ECFP6 | 624 | 700 | 1248 | 25 | 0.961 | 0.641 | 0.721 | 628 | 0 | 1948 | 21 | 0.968 | 1 | 0.992 |
| ^a^TP, true positive; ^b^FP, false positive; ^c^TN, true negative; ^d^FN, false negative; ^e^SE: sensitivity, SE = TP/(TP + FN); ^f^SP (%): specificity, SP = TN/(TN + FP); ^g^Q (%): overall accuracy, Q = (TP + TN)/(TP + FP + TN + FN). | | | | | | | | | | | | | | | | |

**Table S2. The detailed performance of 60 multiple QSAR models by the test set validation using NB and SVM classifiers.**

| **Object** | **Target** | **Finger**  **print** | **NB** | | | | | | | **SVM** | | | | | | |
| --- | --- | --- | --- | --- | --- | --- | --- | --- | --- | --- | --- | --- | --- | --- | --- | --- |
|  |  |  | **TP^a^** | **FP^b^** | **TN^c^** | **FN^d^** | **SE^e^** | **SP^f^** | **Q^g^** | **TP** | **FP** | **TN** | **FN** | **SE** | **SP** | **Q** |
| HIV-1 | CXCR4 | MACCS | 43 | 0 | 132 | 1 | 0.977 | 1 | 0.994 | 43 | 0 | 132 | 1 | 0.977 | 1 | 0.994 |
|  |  | ECFP6 | 43 | 0 | 132 | 1 | 0.977 | 1 | 0.994 | 43 | 0 | 132 | 1 | 0.977 | 1 | 0.994 |
|  | CCR5 | MACCS | 93 | 9 | 335 | 22 | 0.809 | 0.974 | 0.932 | 113 | 1 | 343 | 2 | 0.983 | 0.997 | 0.993 |
|  |  | ECFP6 | 112 | 42 | 302 | 3 | 0.974 | 0.878 | 0.902 | 110 | 0 | 344 | 5 | 0.957 | 1 | 0.989 |
|  | GP120 | MACCS | 73 | 55 | 200 | 12 | 0.859 | 0.784 | 0.803 | 62 | 2 | 253 | 23 | 0.729 | 0.992 | 0.926 |
|  |  | ECFP6 | 73 | 79 | 176 | 12 | 0.859 | 0.69 | 0.732 | 44 | 0 | 255 | 41 | 0.518 | 1 | 0.879 |
|  | GP41 | MACCS | 7 | 0 | 21 | 0 | 1 | 1 | 1 | 7 | 0 | 21 | 0 | 1 | 1 | 1 |
|  |  | ECFP6 | 7 | 0 | 21 | 0 | 1 | 1 | 1 | 7 | 0 | 21 | 0 | 1 | 1 | 1 |
|  | RT | MACCS | 606 | 380 | 2551 | 371 | 0.62 | 0.87 | 0.808 | 911 | 11 | 2920 | 66 | 0.932 | 0.996 | 0.98 |
|  |  | ECFP6 | 832 | 744 | 2187 | 145 | 0.852 | 0.746 | 0.773 | 907 | 5 | 2926 | 70 | 0.928 | 0.998 | 0.981 |
|  | IN | MACCS | 455 | 89 | 1706 | 143 | 0.761 | 0.95 | 0.903 | 575 | 8 | 1787 | 23 | 0.962 | 0.996 | 0.987 |
|  |  | ECFP6 | 520 | 212 | 1583 | 78 | 0.87 | 0.882 | 0.879 | 565 | 3 | 1792 | 33 | 0.945 | 0.998 | 0.985 |
|  | PR | MACCS | 1038 | 82 | 3742 | 236 | 0.815 | 0.979 | 0938 | 1252 | 2 | 3822 | 22 | 0.983 | 0.999 | 0.995 |
|  |  | ECFP6 | 1232 | 392 | 3432 | 42 | 0.967 | 0.897 | 0.915 | 1242 | 1 | 3823 | 32 | 0.975 | 1 | 0.994 |
|  | Gag-pol | MACCS | 29 | 3 | 95 | 3 | 0.906 | 0.969 | 0.954 | 30 | 0 | 98 | 2 | 0.938 | 1 | 0.985 |
|  |  | ECFP6 | 30 | 9 | 89 | 2 | 0.938 | 0.908 | 0.915 | 30 | 0 | 98 | 2 | 0.938 | 1 | 0.985 |
|  | Protein tat | MACCS | 103 | 88 | 320 | 33 | 0.757 | 0.784 | 0.778 | 88 | 11 | 397 | 48 | 0.647 | 0.973 | 0.892 |
|  |  | ECFP6 | 106 | 71 | 337 | 30 | 0.779 | 0.826 | 0.814 | 68 | 1 | 407 | 68 | 0.5 | 0.998 | 0.873 |
|  | PKC | MACCS | 9 | 0 | 29 | 0 | 1 | 1 | 1 | 7 | 0 | 29 | 2 | 0.788 | 1 | 0.947 |
|  |  | ECFP6 | 8 | 0 | 29 | 1 | 0.889 | 1 | 0.974 | 7 | 0 | 29 | 2 | 0.778 | 1 | 0.947 |
|  | CYP3A | MACCS | 5 | 0 | 15 | 0 | 1 | 1 | 1 | 5 | 0 | 15 | 0 | 1 | 1 | 1 |
|  |  | ECFP6 | 5 | 0 | 15 | 0 | 1 | 1 | 1 | 4 | 0 | 15 | 1 | 0.8 | 1 | 0.95 |
| HCV | NS3/4A | MACCS | 92 | 2 | 408 | 44 | 0.676 | 0.995 | 0.916 | 135 | 0 | 410 | 1 | 0.993 | 1 | 0.998 |
|  |  | ECFP_6 | 132 | 32 | 378 | 4 | 0.971 | 0.922 | 0.934 | 128 | 0 | 410 | 8 | 0.941 | 1 | 0.985 |
|  | NS4B | MACCS | 5 | 0 | 20 | 1 | 0.833 | 1 | 0.962 | 5 | 0 | 20 | 1 | 0.833 | 1 | 0.962 |
|  |  | ECFP_6 | 5 | 0 | 20 | 1 | 0.833 | 1 | 0.962 | 5 | 0 | 20 | 1 | 0.833 | 1 | 0.962 |
|  | NS5A | MACCS | 13 | 0 | 40 | 0 | 1 | 1 | 1 | 13 | 0 | 40 | 0 | 1 | 1 | 1 |
|  |  | ECFP_6 | 13 | 0 | 40 | 0 | 1 | 1 | 1 | 13 | 0 | 40 | 0 | 1 | 1 | 1 |
|  | NS5B | MACCS | 218 | 143 | 615 | 35 | 0.862 | 0.811 | 0.824 | 245 | 0 | 758 | 8 | 0.968 | 1 | 0.992 |
|  |  | ECFP_6 | 242 | 288 | 470 | 11 | 0.957 | 0.62 | 0.704 | 247 | 0 | 758 | 6 | 0.976 | 1 | 0.994 |
|  | ^a^TP, true positive; ^b^FP, false positive; ^c^TN, true negative; ^d^FN, false negative; ^e^SE: sensitivity, SE = TP/(TP + FN); ^f^SP (%): specificity, SP = TN/(TN + FP); ^g^Q (%): overall accuracy, Q = (TP + TN)/(TP + FP + TN + FN). | | | | | | | | | | | | | | | |

**Table S3. The prediction results of polypharmacology for 27 approved HIV-1 drugs and 10 approved HCV drugs.**

| **Drugs** | **CXCR4** | **CCR5** | **GP120** | **GP41** | **RT** | **IN** | **PR** | **Gag-pol** | **Protein**  **tat** | **CYP3A** | **NS3/4A** | **NS5A** | **NS5B** |
| --- | --- | --- | --- | --- | --- | --- | --- | --- | --- | --- | --- | --- | --- |
| Enfuvirtide |  |  |  | ++  ++ | + | ++ | ++ |  |  |  |  |  |  |
| Abacavir | + | + | + |  | +++ | + |  | + | + |  |  |  | + |
| Delavirdine | + |  |  |  | ++++ |  |  |  |  |  |  |  | ++ |
| Didanosine |  |  | + |  | ++++ |  |  |  | + |  |  |  | ++ |
| Efavirenz |  |  |  |  | +++ |  |  |  |  |  |  |  | + |
| Emtricitabine |  |  | + |  | ++++ | + |  |  | + |  |  |  | + |
| Etravirine |  |  | + |  | ++++ |  |  |  |  |  |  |  | + |
| Lamivudine |  |  | + |  | ++++ |  |  |  | ++ |  |  |  | + |
| Nevirapine |  |  |  |  | ++++ |  |  |  |  |  |  |  | + |
| Rilpivirine |  |  | + |  | ++ |  |  |  |  |  |  |  | + |
| Stavudine |  |  | + |  | ++++ | + |  |  | ++  + |  |  |  | + |
| Tenofovir disoproxil |  |  | + |  | ++ |  | + | + |  |  |  |  | + |
| Zalcitabine | ++ |  | + |  | ++++ |  |  |  | ++ |  |  |  | + |
| Zidovudine | ++ | ++ | + |  | +++ | + |  | + | + |  |  |  | + |
| Dolutegravir |  | + |  |  | ++ | ++++ |  |  |  |  |  |  | + |
| Elvitegravir |  |  | + |  | ++ | ++++ |  |  |  |  |  |  | ++ |
| Raltegravir |  | + |  |  | + | ++++ |  |  |  |  |  |  | + |
| Amprenavir |  | + | ++ |  | + |  | ++++ | ++  ++ |  |  |  |  | ++ |
| Atazanavir | + | + |  |  | +++ | + | ++++ | + |  |  |  |  | + |
| Darunavir | + | + | ++ |  | + |  | ++++ | ++  ++ |  |  |  |  | ++ |
| Indinavir | + | ++ |  |  | ++ | + | ++++ |  |  |  |  |  | ++ |
| Lopinavir |  | + |  |  | + |  | ++++ | + |  |  |  |  | + |
| Nelfinavir | + | ++  + |  |  | + |  | ++++ | + |  |  |  |  | + |
| Ritonavir |  |  |  |  |  |  | ++++ | + |  | ++  ++ |  |  | + |
| Saquinavir | + | + |  |  | + | + | ++++ | + |  |  |  |  |  |
| Tipranavir |  | + | ++ |  | ++ | + | ++++ | + | + |  |  |  | ++ |
| Cobicistat |  | + |  |  |  |  | +++ | + |  | ++  ++ |  |  |  |
| Boceprevir |  |  |  |  | + |  | + |  |  |  | ++  ++ |  |  |
| Telaprevir |  |  |  |  | + |  | ++ |  |  |  | ++  ++ |  | + |
| Simeprevir |  |  |  |  | ++ |  | ++ |  |  |  | ++  ++ |  | + |
| Grazoprevir |  |  |  |  | + |  | ++ |  |  |  | ++  + |  | ++ |
| Daclatasvir |  |  |  |  |  |  |  |  |  |  | + | ++  ++ |  |
| Ledipasvir |  |  |  |  |  |  | + |  |  |  | + | ++  + | + |
| Elbasvir |  |  |  |  |  |  | + |  |  |  | + | ++  ++ | + |
| Ombitasvir |  |  |  |  |  |  |  |  |  |  | + | ++  + |  |
| Velpatasvir |  |  |  |  |  | + | + |  |  |  | + | ++  + | + |
| Dasabuvir |  |  |  |  | ++ |  | + |  |  |  |  |  | ++ |

**Table S4. 56 chemical-protein interaction pairs predicted by all classifiers.**

| **Drug name** | **Predicted target** | **The NO. of classifiers** | **Validated by refs** |
| --- | --- | --- | --- |
| Enfuvirtide | GP41 | ++++ | True |
| Enfuvirtide | IN | ++ |  |
| Enfuvirtide | PR | ++ |  |
| Delavirdine | RT | ++++ | True |
| Delavirdine | NS5B | ++ |  |
| Didanosine | RT | ++++ | True |
| Didanosine | NS5B | ++ |  |
| Lamivudine | RT | ++++ | True |
| Lamivudine | Protein tat | ++ |  |
| Stavudine | RT | ++++ | True |
| Stavudine | Protein tat | +++ |  |
| Zalcitabine | CXCR4 | ++ |  |
| Zalcitabine | RT | ++++ | True |
| Zalcitabine | Protein tat | ++ |  |
| Zidovudine | CXCR4 | ++ |  |
| Zidovudine | CCR5 | ++ |  |
| Zidovudine | RT | +++ | True |
| Dolutegravir | RT | ++ | false |
| Dolutegravir | IN | ++++ | True |
| Elvitegravir | RT | ++ | True |
| Elvitegravir | IN | ++++ | True |
| Elvitegravir | NS5B | ++ |  |
| Amprenavir | GP120 | ++ |  |
| Amprenavir | PR | ++++ | True |
| Amprenavir | Gag-pol | ++++ |  |
| Amprenavir | NS5B | ++ |  |
| Atazanavir | RT | +++ | True |
| Atazanavir | PR | ++++ | True |
| Darunavir | GP120 | ++ |  |
| Darunavir | PR | ++++ | True |
| Darunavir | Gag-pol | ++++ |  |
| Darunavir | NS5B | ++ |  |
| Indinavir | CCR5 | ++ |  |
| Indinavir | RT | ++ | Inconclusive |
| Indinavir | PR | ++++ | True |
| Indinavir | NS5B | ++ |  |
| Nelfinavir | CCR5 | +++ |  |
| Nelfinavir | PR | ++++ | True |
| Ritonavir | PR | ++++ | True |
| Ritonavir | CYP3A | ++++ | True |
| Tipranavir | GP120 | ++ |  |
| Tipranavir | RT | ++ |  |
| Tipranavir | PR | ++++ | True |
| Tipranavir | NS5B | ++ |  |
| Cobicistat | PR | +++ | True |
| Cobicistat | CYP3A | ++++ | True |
| Telaprevir | PR | ++ |  |
| Telaprevir | NS3/4A | ++++ | True |
| Simeprevir | RT | ++ |  |
| Simeprevir | PR | ++ |  |
| Simeprevir | NS3/4A | ++++ | True |
| Grazoprevir | PR | ++ |  |
| Grazoprevir | NS3/4A | +++ | True |
| Grazoprevir | NS5B | ++ |  |
| Dasabuvir | RT | ++ |  |
| Dasabuvir | NS5B | ++ | True |

**Table S5. The prediction results of polypharmacology for known active compounds toward HIV-1 and HCV.**

| **ChEMBL ID** | **PR** | **IN** | **RT** | **NS5B** |
| --- | --- | --- | --- | --- |
| CHEMBL16326 |  | ++++ |  | ++++ |
| CHEMBL18927 | + | ++++ | + | ++++ |
| CHEMBL449221 |  | ++++ | + | ++++ |
| CHEMBL502238 |  | ++++ |  | ++++ |
| CHEMBL19332 |  | ++++ | + | ++++ |
| CHEMBL210593 |  |  | ++ | +++ |
| CHEMBL37541 | ++++ |  | + | ++ |
| CHEMBL3612421 | ++++ | + |  | ++ |
| CHEMBL1668670 | + | ++++ | ++++ | ++ |

**Table S6. The docking score of 9 selected compounds and 6 drugs that towards HIV-1 PR, RT, IN and HCV NS5B.**

| Ligand | Target | Docking score |
| --- | --- | --- |
| Elvitegravir | IN | -5.56 |
| Stavudine | RT | -8.37 |
| Nelfinavir | PR | -9.36 |
| Sofosbuvir | NS5B (Catalytic Site) | -7.85 |
| MK3281^a^ | NS5B (Thumb Site I) | -7.88 |
| Filibuvir^b^ | NS5B (Thumb Site II) | -6.66 |
| CHEMBL16326 | IN | -6.41 |
| CHEMBL16326 | NS5B (Catalytic Site) | -9.45 |
| CHEMBL18927 | IN | -6.22 |
| CHEMBL18927 | NS5B (Catalytic Site) | -7.83 |
| CHEMBL449221 | IN | -6.78 |
| CHEMBL449221 | NS5B (Catalytic Site) | -9.90 |
| CHEMBL502238 | IN | -6.60 |
| CHEMBL502238 | NS5B (Catalytic Site) | -9.57 |
| CHEMBL19332 | IN | -6.59 |
| CHEMBL19332 | NS5B (Catalytic Site) | -9.93 |
| CHEMBL210593 | RT | -8.25 |
| CHEMBL210593 | NS5B (Thumb Site I) | -5.05 |
| CHEMBL37541 | PR | -7.61 |
| CHEMBL37541 | NS5B (Thumb Site II) | --^c^ |
| CHEMBL3612421 | PR | --^d^ |
| CHEMBL3612421 | NS5B (Thumb Site II) | -7.59 |
| CHEMBL1668670 | PR | --^d^ |
| CHEMBL1668670 | NS5B (Thumb Site II) | -8.60 |
| ^a^MK3281 is a Thumb Site I nonnucleoside inhibitor of NS5B; ^b^Filibuvir is a Thumb Site II nonnucleoside inhibitor of NS5B; ^c^The crystal structure of CHEMBL37541 in complex with NS5B; ^d^No docking study. | | |
